# Historically Black College or University Attendance and Cognition in US Black Adults

**DOI:** 10.1001/jamanetworkopen.2025.58329

**Published:** 2026-02-11

**Authors:** Marilyn D. Thomas, Carol Wei, Min Hee Kim, Jennifer Manly, Suzanne E. Judd, Justin S. White, Virginia J. Howard, Christina Mangurian, Rita Hamad, M. Maria Glymour

**Affiliations:** 1 Division of General Internal Medicine, Department of Medicine, University of California, San Francisco; 2 Department of Epidemiology and Biostatistics, University of California, San Francisco; 3 Philip R. Lee Institute for Health Policy Studies, University of California, San Francisco; 4 Center for Health Services Research, Institute for Health, Health Care Policy and Aging, School of Nursing, Rutgers University, New Brunswick, New Jersey; 5 Department of Psychiatry and Behavioral Sciences, University of California, San Francisco; 6 Department of Health Behavior, School of Public Health, University of Alabama at Birmingham, Birmingham; 7 Columbia University Irving Medical Center, New York, New York; 8 Department of Health Law, Policy, & Management, Boston University School of Public Health, Boston, Massachusetts; 9 Department of Epidemiology, School of Public Health, University of Alabama at Birmingham, Birmingham; 10 Department of Social and Behavioral Sciences, Harvard T.H. Chan School of Public Health, Boston, Massachusetts; 11 Department of Epidemiology, Boston University School of Public Health, Boston, Massachusetts

## Abstract

**Importance:**

Black adults may derive long-term cognitive benefits from attendance at a historically Black college or university (HBCU) compared with a predominantly White institution (PWI). This association has not been evaluated in a nationwide sample.

**Objective:**

To estimate the association between HBCU vs PWI attendance and cognitive health among later-life Black adults.

**Design, Setting, and Participants:**

This cohort study used data from the Reasons for Geographic and Racial Differences in Stroke study, a prospective cohort study that recruited Black and White US adults aged 45 years and older during 2003 to 2007. The national cohort oversampled Black individuals and residents from the Stroke Belt (56%), a group of 8 Southern states defined by excess stroke mortality. The analytic sample included Black participants who attended high school in a state with an HBCU and attended college. Analysis was conducted from February to September 2025.

**Exposure:**

Participants self-reported each college ever attended, classified as a PWI (reference) or HBCU.

**Main Outcomes and Measures:**

Assessments of memory, language, and global cognition were conducted during follow-up every 6 months (2006-2021). Cognitive measures were standardized (ie, *z*-transformed). Linear regression was used to estimate the average treatment effect (ATE) using inverse of probability of treatment weighting. Additional analyses evaluated potential modification by being college-aged before 1955 (during legal racial segregation), 1955 to 1964 (during sanctioned racial discrimination), or after 1964.

**Results:**

Among 1978 Black college-goers (mean [SD] age at first assessment, 61.8 [8.2] years; 1333 [67.4%] female), 699 (35.3%) attended an HBCU, 1952 (98.7%) completed a memory assessment, 1970 (99.6%) completed a language assessment, and 530 (26.8%) completed both assessments during the same follow-up visit. Compared with PWI attendance, HBCU attendance was associated with better *z*-scored memory (ATE, 0.13; 95% CI, 0.05-0.21), language (ATE, 0.19; 95% CI, 0.08-0.29), and global cognition (ATE, 0.22; 95% CI, 0.09-0.34). Estimates were consistent for individuals who were college-aged after 1955 but were not statistically significant among respondents who were college-aged prior to 1955.

**Conclusions and Relevance:**

In this cohort study using a national dataset, HBCU attendance was associated with better cognition compared with PWI attendance for aging Black adults, which held for those attending college before and after legal racial segregation and sanctioned racial discrimination in education.

## Key Points

**Question:**

Is attending a historically Black college or university (HBCU) vs a predominantly White institution (PWI) associated with later-life cognitive health for Black adults in the US?

**Findings:**

In this cohort study of 1978 Black college-goers who were college-aged circa 1940 to 1980, HBCU attendance was associated with better *z*-scored memory, language, and global cognition compared with PWI attendance.

**Meaning:**

These findings suggest HBCU attendance may have long-term cognitive benefits for Black adults that are robust to historical and ongoing inequities resulting from racialized education policies.

## Teaser

This cohort study examines the association of attending a historically Black college or university compared with a predominantly White institution with cognitive health in later life among Black adults.

## Introduction

Considerable evidence identifies higher education as a strong social determinant of reduced risk of Alzheimer disease and related dementias (ADRD)^1,2,3^; however, inequities in ADRD persist between Black and White college-educated individuals.^4^ People with higher education are thought to have greater cognitive reserve,^5,6,7^ described as preserved cognitive function despite measures of neuropathology and diminished brain integrity. However, a growing body of evidence demonstrates that higher education may not offer equal cognitive reserve protection against ADRD risk for Black adults compared with White adults,^8,9^ particularly in the memory and language cognitive domains.^8^ To address this disparity, it is imperative to consider educational sources of resilience that impact Black adults differentially than the general population. Historically Black colleges and universities (HBCUs) offer a promising possible example.

HBCUs were generally established after the US Civil War (circa 1860) to provide Black US residents with postsecondary education at a time when Black people were excluded from institutions of higher learning.^10^ Even with the landmark 1954 *Brown v Board of Education of Topeka* Supreme Court ruling that segregated public schools were unconstitutional, US educational experiences continue to be racially segregated, with predominantly Black schools grossly underresourced.^11,12^ Titles VII and XI of the 1964 Civil Rights Act expanded access to public higher education for historically excluded groups, including some additional, albeit modest, federal funding for HBCUs.^13^

Despite being historically underfunded,^14,15^ HBCUs continue to primarily enroll Black students (76%),^15^ in part because a central focus of HBCUs is to foster Black culture, pride, and excellence by promoting Black scholarship, alumni networks, high-quality education, and student success. HBCUs have gone on to uplift Black individuals as well as Black communities through economic and cultural empowerment, increased social and political capital, and neighborhood revitalization.^16,17^ As such, federal resources have been reallocated recently to enhance the success and effectiveness of HBCUs,^18,19^ creating an opportunity to grow our understanding of the health implications of HBCU attendance.

Promising new evidence suggests that attending an HBCU vs a predominantly White institution (PWI) may improve later-life cognition for Black adults. Using data from the National Longitudinal Study of Adolescent to Adult Health study, Colen et al^20^ found that Black HBCU (vs PWI) attendees had lower risk of developing metabolic syndrome, a cluster of metabolic disturbances associated with ADRD progression.^21,22^ In a more recent study, Thomas et al^23^ reported a positive association of HBCU attendance with cognitive outcomes using data from middle-aged and older health care system members in Northern California, although estimates were imprecise. A deeper examination in a national study of Black adults is needed.

**Figure 1.**
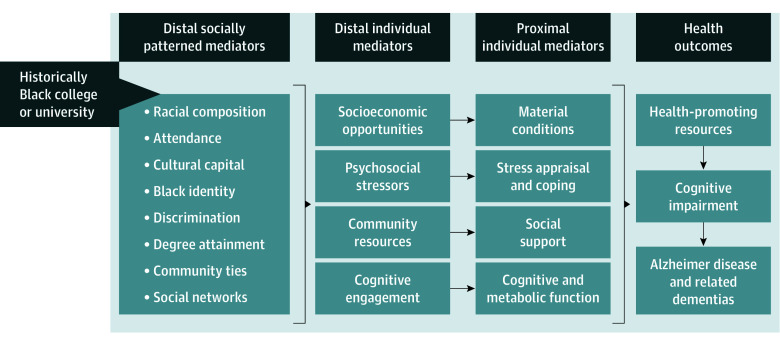
Theoretical Pathway Linking Attendance at a Historically Black College or University to Cognitive Impairment Adapted from Glymour and Manly,^24^ 2008.

Guided by fundamental cause and life course theories,^24,25^ we identified possible pathways by which HBCU exposure may influence cognition that include cultural capital, interpersonal discrimination, and community resources that determine individual-level exposures to psychological stressors and cognitive engagement for Black students (Figure 1). Among older US Black adults who attended college, we estimated the association of attending an HBCU vs a PWI with cognitive function. We hypothesized that compared with PWI attendance, HBCU attendance would be associated with better memory, language, and global cognition for aging Black adults.

## Methods

This cohort study was conducted as part of the Reasons for Geographic and Racial Differences in Stroke (REGARDS) study. The institutional review boards of participating institutions approved all REGARDS study methods. Informed consent was obtained verbally by telephone and later in writing during the in-person evaluation. All study procedures adhere to the Strengthening the Reporting of Observational Studies in Epidemiology (STROBE) reporting guideline.

### Study Participants

The REGARDS study is a national cohort of more than 30 000 Black and White adults recruited during 2003 to 2007 at age 45 years or older. By design, the cohort oversampled Black individuals and residents from the so-called Stroke Belt (56%), a group of 8 Southern states defined by excess stroke mortality.^26^ At baseline, using a computer-assisted telephone interview, trained interviewers obtained demographic information, medical history, and lifestyle factors, including a selection of potential risk factors. During an in-person assessment, self-administered questionnaires were left with the participant to be returned by addressed, prepaid envelopes. One questionnaire included residential history information recording city and state (or country) of birth, and every city and state where the participant had lived for at least 1 year, and age at which they moved from each location. Detailed methods of coding and classification of places lived and age are described elsewhere.^27^ Every 6 months, participants were interviewed by computer-assisted telephone interview for cognitive assessments. In 2012, an ancillary study was initiated to collect childhood and family life factors through a mail questionnaire to all active participants.

**Figure 2.**
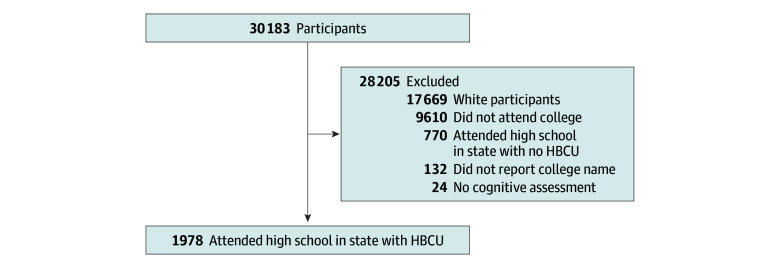
Process of Sample Selection Among Reasons for Geographic and Racial Differences in Stroke Study Participants Adapted from the Reasons for Geographic and Racial Differences in Stroke study website.^28^ HBCU indicates historically Black college or university.

Among 12 514 Black participants, 2904 (23.2%) attended college (Figure 2).^28^ Among 2772 participants (95.5%) reporting the name of the college attended, 2748 (99.1%) completed a memory or language assessment. Given that HBCUs are located primarily in the South and Washington, District of Columbia, we restricted our analytic sample to 1978 participants who attended a high school in a state with an HBCU, including the District of Columbia, and would thereby have similar probabilities of HBCU attendance. Because restricting the analysis to HBCU states also restricts estimate precision and assumes that all college attendees were comparable regardless of confounding factors or selection into the study, we conducted several sensitivity analyses to assess the robustness of our methods.

### Exposure Measure

There are 107 established HBCUs in the US.^15,29^ After performing a comprehensive cleaning of all college names, participant residential history was used to confirm each HBCU location by county and state. Each HBCU was further verified using Integrated Postsecondary Education Data System codes.^30^ A dichotomous variable was generated to indicate whether the participant had ever attended an HBCU (0 = PWI; 1 = HBCU), regardless of degree attainment.

### Outcome Measures

#### Memory

Memory scores were derived from immediate recall and delayed recall word list learning tests taken from the Consortium to Establish a Registry for Alzheimer Disease battery.^31^ Immediate recall involved 3 trials of 10 words; scores on 3 trials were summed for a raw score ranging from 0 to 30. Delayed recall involved repeating as many words as one could remember from the immediate assessment. The delayed recall raw score was rescaled for comparability with immediate recall (range, 0-30). We calculated the mean from the 2 recall tests and converted to *z* scores using means and SDs, with higher scores reflecting better memory.

#### Language

Language scores were obtained using component tasks of semantic animal fluency and phonemic letter *f* fluency. Animal fluency involved naming as many animals as one could think of within a 45-second period (score, 0-59). Letter *f* fluency involved naming as many words beginning with the letter *f* within 60 seconds (range, 0-49). We calculated the mean of the 2 fluency scores and converted to z scores using means and SDs, with higher scores reflecting better language.

#### Global Cognition

We calculated the means for memory and language *z* scores to create a global composite measure, with higher scores reflecting better global cognition. Memory and language assessments could have been taken at different follow-up visits during 2006 to 2021. Therefore, estimates were obtained separately at first assessment, and global cognition estimates were obtained among those who had both assessments taken during the same visit.

### Covariates

Both theoretical and data-driven approaches were used to select covariates to minimize systematic bias and maximize statistical power by fitting the most parsimonious model. We used a directed acyclic graph to map out hypothesized causal pathways expected to differentially influence the likelihood of HBCU attendance for Black adults who attended high school near an HBCU and the risk of cognitive impairment in later-life (eFigure 1 in Supplement 1). In addition to participant characteristics, 5 childhood domains were observed in REGARDS: individual-level socioeconomic status, health, academic support, and social support, as well as state-level exposures known to disproportionately constrain the power, resources, and opportunities of Black individuals. Because REGARDS has multiple indicators within each domain, we first performed bivariable analyses to assess the strength of associations between independent and dependent variables at α = .10 (eTable 1 and eTable 2 in Supplement 1). Then, the strongest confounders were assessed for best model fit using forward stepwise regression and nested regression using Schwarz bayesian information criterion.

Our final models were adjusted for age at cognitive assessment (centered at mean age 62 years), gender (0 = female; 1 = male), college-aged birth cohort (0 = pre-*Brown* [<1955]; 1 = post-*Brown* to pre-Civil Rights Act [CRA; 1955-1964]; 2 = post-CRA [>1964]), community size at birth (0 = city or large town; 1 = rural or small town), and, during childhood (ages 14-19 years), mother or female caregiver’s education (0 = college; 1 = completed high school; 2 = <high school and unknown), general health (0 = excellent or very good; 1 = good, fair, or poor), being shown love and affection (0 = all the time; 1 = some or most of the time; 2 = little, none, or unknown), encouragement to succeed in school (0 = all the time; 1 = some or most of the time; 2 = little, none, or unknown), and the mean state-level percentage of the population at or below the federal poverty level across the decades of participant college years (1940-1980).

### Statistical Analysis

All analyses were conducted using Stata software version 18.0 (StataCorp). Frequencies and central tendencies described participant characteristics. We used χ^2^ and *t* tests to evaluate differences between HBCU and PWI attendees. Extreme outliers (>3 SDs from each outcome mean) were also identified.

Multiple imputation by chained equations (*m* = 50) was used to handle missing data (<6% participant data; 10% state-level data).^32^ Following best practices,^32,33^ imputations were obtained using both independent and outcome variables, and then observations with imputed outcomes were deleted before the analysis.

Using the imputed data, we used inverse probability of treatment weights (IPTWs) to help address residual confounding by estimating each participant’s inverse probability of receiving an HBCU treatment assignment, given their set of covariates.^34^ IPTWs render HBCU attendance statistically independent of the measured confounders in the reweighted population. We used inverse probability–weighted regression adjustment (IPWRA), a doubly robust estimator that combines IPTW (treatment modeling) and regression adjustment (outcome modeling), to obtain correct estimates if the models were misspecified. The weights were generated using continuous age and state-level covariates; however, all remaining covariates were discretized into categories, which should help account for nonlinear associations and negate the need for higher-order terms. The teffects ipwra command with the cmdok option calculated the weights and used the weighted regression coefficients to compute means of HBCU estimated outcomes (Y). Then, the means were used to estimate the average treatment effect (ATE), the expected difference in the probability across the entire population if all Black college-goers attended an HBCU (Y_1_) vs if none attended an HBCU (Y_0_): *ATE* = *E*(*Y_1_*) – *E*(*Y_0_*). Given that postestimation commands are unsupported using imputed data,^35^ assessment of the weighted covariates was conducted in adjusted models prior to imputation using teffects overlap, tebalance summarize, tebalance overid, and tebalance density.

Linear regression was used to estimate a 1-SD change in the *z*-scored cognitive outcomes. We clustered SEs by participants’ high school state (n = 20) using vce(cluster) to account for correlated outcomes due to the shared environment of those attending high school in the same state. In the primary analyses, the sample was restricted to respondents who attended high school in a state with an HCBU (eTable 3 in Supplement 1). To ensure the robustness of findings, we performed sensitivity analyses to the IPTW models that included (1) Black college-goers in all states (ie, not just HBCU states), (2) state-fixed effects, (3) adjusting for state-level Black population, (4) omitting state-level covariates, and (5) omitting extreme outliers. Furthermore, we conducted generalized linear regression without IPTW for those in HCBU states, with and without state-fixed effects, and among those living in all states.

Because study participants were at college age before and after legal racial segregation (1954) and the CRA prohibiting racial discrimination in education (1964), we conducted additional analyses to detect potential modification by college-aged birth cohort using an HBCU × birth cohort interaction term.

*P* values were 2-sided, and statistical significance was set at *P* ≤ .05. Analysis was conducted from February 2025 to September 2025.

## Results

### Participant Characteristics

Among 1978 Black college-goers (mean [SD] age at first assessment, 61.8 [8.2] years; 1333 [67.4%] female), 699 (35.3%) attended an HBCU. The distribution of participant characteristics was similar across assessment groups (Table), hence group means are described. Compared with PWI attendees, during childhood, HBCU attendees were more likely to have a mother or female caregiver with a college education (135 participants [19.3%] vs 109 participants [8.5%]), encouragement to succeed in school (504 participants [72.1%] vs 721 participants [57.4%]), have been shown love and affection (467 participants [66.8%] vs 777 participants [60.8%]), and live in a state with a larger Black population (25.3% vs 21.5%) and greater poverty (22.0% vs 19.4%). The memory score distribution had no outliers, while language had 23 outliers and global cognition had 5 outliers.

**Table.** Characteristics of Participants Who Attended High School in a State With an HBCU by First Cognitive Assessment and Type of College Attended

Variable	Participants								
	Memory (n = 1952)			Language (n = 1970)			Global composite (n = 530)		
	No. (%)		*P* value	No. (%)		*P* value	No. (%)		*P* value
	HBCU (n = 691)	PWI (n = 1261)		HBCU (n = 699)	PWI (n = 1271)		HBCU (n = 170)	PWI (n = 360)	
Assessment age, mean (SD), y	66.7 (8.2)	65.5 (8.6)	.004	66.7 (8.2)	65.4 (8.5)	<.001	65.9 (8.8)	64.7 (8.5)	.12
Gender									
Male	232 (33.6)	406 (32.2)	.54	233 (33.3)	408 (32.1)	.58	64 (37.6)	109 (30.3)	.091
Female	459 (66.4)	855 (67.8)		466 (66.7)	863 (67.9)		106 (62.4)	251 (69.7)	
College age									
Pre-*Brown* (<1955)	204 (29.5)	309 (24.5)	.009	204 (29.2)	312 (24.6)	.95	39 (23.0)	64 (17.8)	.16
Post-*Brown* to pre-CRA (1955-1964)	316 (45.7)	568 (45.0)		323 (46.2)	571 (44.9)		74 (43.5)	147 (40.8)	
Post-CRA (>1964)	171 (24.8)	384 (30.5)		172 (24.6)	388 (30.5)		57 (33.5)	149 (41.4)	
Community size at birth									
City or large town	245 (35.5)	444 (35.2)	.92	249 (35.6)	449 (35.3)	.95	56 (32.9)	137 (38.1)	.51
Rural or small town	419 (60.6)	772 (61.2)		423 (60.5)	776 (61.0)		110 (64.7)	214 (59.4)	
Missing	27 (3.9)	45 (3.6)		27 (3.9)	46 (3.6)		4 (2.4)	9 (2.5)	
Mother’s or female caregiver’s education									
College	135 (19.5)	109 (8.6)	<.001	135 (19.3)	109 (8.6)	<.001	30 (17.6)	32 (8.9)	.02
Post–high school vocational/professional	44 (6.4)	74 (5.9)		44 (6.3)	74 (5.8)		11 (6.5)	25 (6.9)	
Hight school or GED	100 (14.5)	183 (14.5)		103 (14.7)	183 (14.4)		29 (17.1)	48 (13.3)	
<High school, none, or unknown	405 (58.6)	877 (69.5)		410 (58.7)	887 (69.8)		98 (57.7)	247 (68.6)	
Missing	7 (1.0)	18 (1.4)		7 (1.0)	18 (1.4)		2 (1.2)	8 (2.2)	
Childhood encouragement to succeed in school									
All the time	497 (71.9)	712 (56.5)	<.001	504 (72.1)	720 (56.6)	<.001	126 (74.1)	203 (56.4)	<.001
Most of the time	92 (13.3)	206 (16.3)		93 (13.1)	207 (16.3)		19 (11.2)	59 (16.4)	
Some of the time	36 (5.2)	130 (10.3)		36 (5.1)	131 (10.3)		9 (5.3)	36 (10.0)	
Little of time	22 (3.2)	90 (7.1)		22 (3.1)	88 (6.9)		5 (2.9)	28 (7.8)	
None of the time	10 (1.4)	68 (5.4)		10 (1.4)	70 (5.5)		3 (1.8)	23 (6.4)	
Missing	34 (4.9)	55 (4.4)		34 (4.9)	55 (4.3)		8 (4.7)	11 (3.1)	
Childhood general health									
Excellent or very good	481 (69.6)	858 (68.0)	.35	488 (69.8)	863 (67.9)	.30	130 (76.5)	251 (69.7)	.18
Good, fair, or poor	188 (27.2)	373 (29.6)		189 (27.0)	378 (29.7)		36 (21.2)	103 (28.6)	
Missing	22 (3.2)	30 (2.4)		22 (3.2)	30 (2.4)		4 (2.3)	6 (1.7)	
Shown childhood love and affection									
All the time	460 (66.6)	766 (60.8)	.002	467 (66.8)	773 (60.8)	.001	113 (66.5)	228 (63.3)	.02
Some or most the time	168 (24.3)	343 (27.2)		169 (24.2)	341 (26.8)		43 (25.3)	83 (23.1)	
Little, none, or unknown time	30 (4.3)	104 (8.2)		30 (4.3)	108 (8.5)		5 (2.9)	38 (10.6)	
Missing	33 (4.8)	48 (3.8)		33 (4.7)	49 (3.9)		9 (5.3)	11 (3.1)	
State-level Black population, mean (SD), %^a^	25.2 (10.4)	21.5 (10.6)	<.001^b^	25.3 (10.3)	21.5 (10.5)	<.001^b^	26.1 (9.9)	23.0 (9.7)	.002^c^
State-level population below the FPL, mean (SD), %^a^	21.9 (6.4)	19.4 (6.3)	<.001^b^	21.9 (6.4)	19.5 (6.3)	<.001^b^	22.8 (5.6)	20.1 (5.6)	<.001^c^

Abbreviations: CRA, Civil Rights Act; FPL, Federal Poverty Line; GED, general education diploma; HBCU, historically Black college or university; PWI, predominantly White institution.

^a^ Mean state-level measure across high school decades (1940-1980).

^b^ Sample sizes: HBCU: 615 participants (36.4%); PWI: 1075 participants (63.6%); total: 1690 participants (100%).

^c^ Sample sizes: HBCU: 151 participants (32.7%); PWI: 311 participants (67.3%); total: 462 participants (100%).

### Adjusted Differences

**Figure 3.**
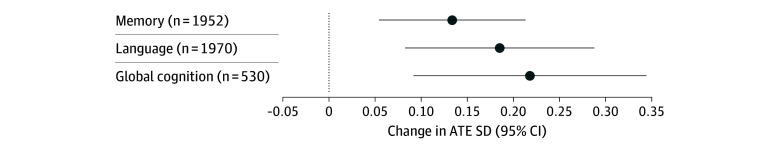
Difference in Cognitive Scores Among Black Participants Who Attended High School in a State With and Attended a Historically Black College or University (vs Attended a Predominantly White Institution) Changes are the SD unit change in the average treatment effect (ATE) with 95% CIs (whiskers).

Black college-goers who attended an HBCU had better mean cognition scores than PWI attendees (Figure 3). HBCU attendance was associated with better memory (ATE, 0.13; 95% CI, 0.05-0.21), language (ATE, 0.19; 95% CI, 0.08-0.29), and global cognition (ATE, 0.22; 95% CI, 0.09-0.34) (eTable 4 in Supplement 1). Estimates from sensitivity analyses were robust to alternative model specifications using IPTW and linear regression: no meaningful differences in estimate magnitude, direction, or precision were observed (eTable 5 in Supplement 1). Postestimation results showed that the estimated propensity scores provided good overlap of covariates between HBCU and PWI attendees in the reweighted population (eFigure 2 in Supplement 1).

### Modification by College-Aged Period

**Figure 4.**
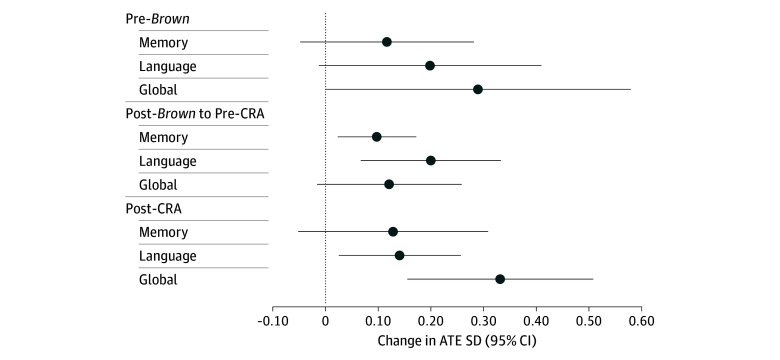
Difference in Cognitive Scores Among Black Participants Who Attended High School in a State With and Attended a Historically Black College or University vs Attended a Predominantly White Institution, Stratified by Period of College Attendance Pre-*Brown* indicates the period prior to the *Brown v Board of Education of Topeka* Supreme Court decision ending legal racial segregation of schools (<1955); Post-*Brown* to pre-CRA, the period prior to the Civil Rights Act (CRA) prohibiting racial discrimination in education (1955-1964); post-CRA, after the adoption of the CRA (>1964). Changes are the SD unit change in the average treatment effect (ATE) with 95% CIs (whiskers).

Interaction with HBCU attendance was detected within strata of college-aged exposure to education policies for all outcomes, hence stratified estimates are reported (eTable 4 in Supplement 1). Compared with PWI attendees, HBCU attendees had better mean cognition scores (Figure 4). College-aged attendees during 1955 to 1964 had better memory (ATE, 0.10; 95% CI, 0.02-0.17) and language (ATE, 0.20; 95% CI, 0.07-0.33). Associations among individuals who attended college after 1964 were attenuated for language (ATE, 0.14; 95% CI, 0.03-0.26) yet higher for global cognition (ATE, 0.33; 95% CI, 0.16-0.51).

## Discussion

To our knowledge, this cohort study is the first national study to examine the association of HBCU vs PWI attendance with later-life cognition in Black adults. Among 1978 middle- to later-life Black college-goers from states with an HBCU, 35% attended an HBCU. At a mean age of 62 years, we found that HBCU attendees had better memory, language, and global cognition. This association was generally similar among subgroups stratified by participants who were college-aged before and after state-sanctioned racial segregation and discrimination in education.

Extensive evidence suggests associations of higher educational attainment with lower ADRD risk and identifies marked disparities between Black and White individuals, yet few studies have evaluated the association of attendance at an HBCU with cognitive risk. Much of the HBCU and health literature focuses on behaviors (eg, sexual activity, substance use, violence, help-seeking, exercise), stress and coping (eg, John Henryism, Superwoman Schema), mental or psychological well-being (eg, depression, distress), racial identity (eg, race centrality, cultural misorientation), and ideology (eg, Black Nationalism).^36,37,38,39,40,41,42,43,44,45,46,47,48,49^ Notably, the estimates for cognition in our study were comparable to prior works examining racialized experiences related to schooling.^50,51,52,53^ For example, attending a segregated vs desegregated school in sixth grade has been associated with lower *z* scores of executive function (*z* score, −0.18; 95% CI, −0.34 to −0.02) and semantic memory (*z* score, −0.31; 95% CI, −0.48 to −0.13).^50^

For Black college students, HBCU exposure may help mitigate the later-life adverse health effects related to de jure and de facto racial segregation (1954) and discrimination in education (1964). In the US South, Jim Crow laws created social and economic inequalities in schooling, housing, labor, and health care that unfairly disadvantage Black individuals over the life course.^54^ As a result, legal school segregation has been linked to poor health outcomes,^25,55,56^ including cognition.^50,51,52^ Moreover, the enforcement of the 1964 Civil Rights Act is associated with improved health among Black individuals and communities.^57^ Consistent with other work,^50,51,52,53^ we found evidence of modification by exposure to racialized education policies. Since HBCU attendees had better cognition than PWI attendees across all birth cohorts and cognitive domains, our finding may be due to chance. Nonetheless, this evidence suggests that the long-term benefits of HBCU attendance may be robust to historical and ongoing inequities resulting from racialized education policies, warranting deeper study using larger sample sizes and additional health outcomes.

HBCU attendance may improve cognitive health through biopsychosocial mechanisms over the life course. Compared with national estimates, HBCU students report lower rates of depression and anxiety and higher rates of positive mental health,^44^ which may improve the benefits of cognitive reserve. Conversely, Black students attending PWIs often report experiences of anti-Black bias and discrimination.^58,59^ Psychosocial stressors can provoke inflammation and oxidative stress,^60^ which can contribute to ADRD development through cerebrovascular inflammatory mechanisms over the life course.^61^ Moreover, Black students at HBCUs (vs PWIs) develop a greater sense of belonging, build stronger social networks, and engage in healthier behaviors to cope with stressors.^36,44,46^ Therefore, additional studies should assess whether HBCU attendance may improve ADRD outcomes and inequities by increasing health-promoting resources and reducing vascular risk of ADRD.

Another notable observation that emerged was the likely influence of personal and academic empowerment during childhood, particularly by caregivers, on the probability of attending an HBCU. Among myriad motivations to choose an HBCU, Black students are strongly influenced by familial perceptions of HBCUs, institutional legacy (eg, schools of parents, friends), and encouragement by trusted supporters (eg, teachers, counselors).^62,63,64,65,66^ Similarly, we found strong HBCU associations with childhood academic and social support, as well as having a college-educated mother or female caregiver. A 2025 qualitative study among educated, higher socioeconomic status Black parents on their views of their college-bound children selecting HBCUs reported insights also consistent with our findings.^67^ For instance, parents had concerns about their children navigating “White spaces” and prioritized the maintenance of their children’s Black identity in preparation. Many parents who attended an HBCU viewed HBCUs as a type of sociocultural preparation, racial socialization, and community building opportunity beyond the academic to ready their children for adult life.^67^ Hence, the powerful impact of Black caregiver love, affection, and academic encouragement may be uniquely relevant for Black HBCU attendees. Additional work should investigate the direct effects of early-life sociocultural and academic encouragement on cognition in later-life Black adults.

There are several notable study considerations. REGARDS recruited a nonrepresentative national sample of Black adults. However, REGARDS is a unique dataset with retrospective childhood factors and residential history. IPWRA is a doubly robust estimator that obtains correct estimates when the regression model is misspecified, which was supported by the results from our sensitivity analyses. Furthermore, the exchangeability of our HBCU and PWI attendees was reinforced by comparable estimates across models using IPTW and generalized linear regression. Accounting for early-life factors also helped minimize selection bias from left truncation or loss to follow-up and increased internal validity. Importantly, HBCU attendance is a novel method to operationalize Black educational experiences as a source of resilience against a disproportionate risk of ADRD in health equity research.

### Limitations

This study has some limitations. Our study findings are not generalizable to the US population, and our cross-sectional design precludes causal interpretation. Other limitations include possible recall bias, measurement error in cognitive assessments (eg, assessment mode) and historical census data, and unmeasured confounding. Additionally, an HBCU measure does not directly capture early-life exposure to culturally relevant institutional characteristics (eg, Black scholarship, political capital, discrimination).

## Conclusions

In this national cohort study of Black college-goers, we found that HBCU attendance was associated with better cognition for aging adults, an association that held for those attending school before the CRA. Applying methods to examine intersectional social positions (eg, gender, socioeconomic factors, academic achievements) would help understand whether individual-level factors modify the association found in this study. Examining other HBCU aspects (eg, prestige, faculty racial composition, funding) could also help elucidate how HBCUs may differentially advantage Black adults. Furthermore, nationally representative panel studies on cognition could help us to understand HBCU impacts on cognitive decline for Black Americans.

## Notes

Supplement 1. **eFigure 1.** Directed acyclic graph representing working knowledge of potential confounders linking attendance at an Historically Black College or University (HBCU) and later-life cognition **eFigure 2.** Postestimation covariate balance results prior to multiple imputation **eTable 1.** Associations between covariates within each childhood domain **eTable 2.** Bivariable associations of covariates with memory, language, and HBCU status **eTable 3.** State or region with a historically Black college or university (HBCU) **eTable 4.** Primary estimates of differences between HBCU vs PWI attendees on cognition at mean age 62 among Black adults in REGARDS, and estimates stratified by college-aged exposure to legal racial segregation (Pre-Brown) and Civil Rights Act (CRA) **eTable 5.** Estimates of the association between HBCU vs PWI attendance on cognition among Black adults at mean age 62 in REGARDS

Supplement 2.
Data Sharing Statement
