# Supplementary material for: Historically Black College or University Attendance and Cognition in US Black Adults
Source: JAMA Netw Open. 2026 Feb 11;9(2):e2558329. doi: 10.1001/jamanetworkopen.2025.58329 (PMC12895292; doi:10.1001/jamanetworkopen.2025.58329)
Supplement: Supplement 1. — eFigure 1. Directed acyclic graph representing working knowledge of potential confounders linking attendance at an Historically Black College or University (HBCU) and later-life cognition eFigure 2. Postestimation covariate balance results prior to multiple imputation eTable 1. Associations between covariates within each childhood domain eTable 2. Bivariable associations of covariates with memory, language, and HBCU status eTable 3. State or region with a historically Black college or university (HBCU) eTable 4. Primary estimates of differences between HBCU vs PWI attendees on cognition at mean age 62 among Black adults in REGARDS, and estimates stratified by college-aged exposure to legal racial segregation (Pre-Brown) and Civil Rights Act (CRA) eTable 5. Estimates of the association between HBCU vs PWI attendance on cognition among Black adults at mean age 62 in REGARDS [file jamanetwopen-e2558329-s001.pdf]

Supplemental Online Content

Thomas MD, Wei C, Kim MH, et al. Historically Black college or university attendance and cognition in US Black adults. *JAMA Netw Open*. 2026;9(2):e2558329.
doi:10.1001/jamanetworkopen.2025.58329

eFigure 1. Directed acyclic graph representing working knowledge of potential confounders linking attendance at an Historically Black College or University (HBCU) and later-life cognition
eFigure 2. Postestimation covariate balance results prior to multiple imputation
eTable 1. Associations between covariates within each childhood domain
eTable 2. Bivariable associations of covariates with memory, language, and HBCU status
eTable 3. State or region with a historically Black college or university (HBCU)
eTable 4. Primary estimates of differences between HBCU vs PWI attendees on cognition at mean age 62 among Black adults in REGARDS, and estimates stratified by college-aged exposure to legal racial segregation (Pre-Brown) and Civil Rights Act (CRA)
eTable 5. Estimates of the association between HBCU vs PWI attendance on cognition among Black adults at mean age 62 in REGARDS

This supplemental material has been provided by the authors to give readers additional information about their work.

© 2026 Thomas MD et al. *JAMA Network Open*.

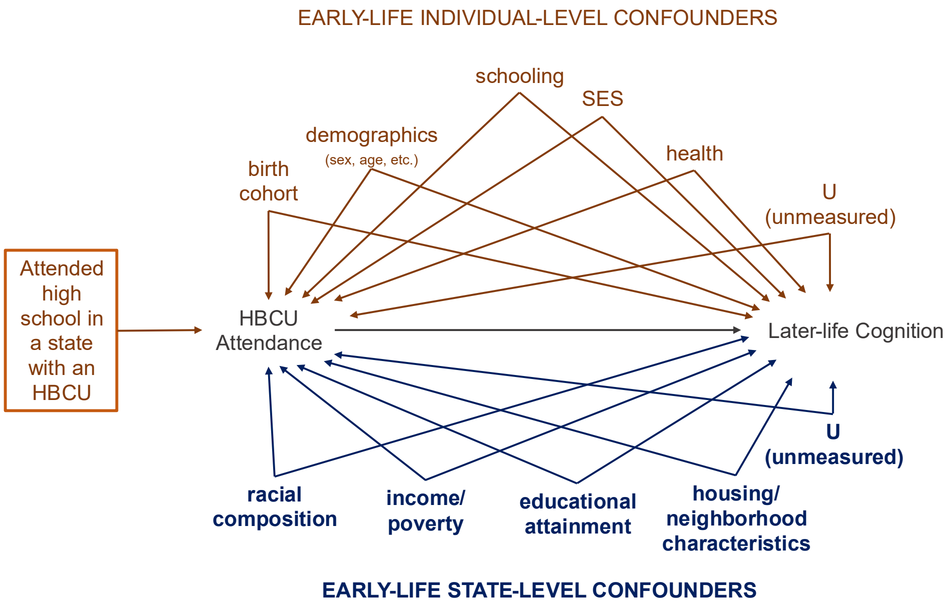

eFigure 1. Directed acyclic graph representing working knowledge of potential confounders linking attendance at an Historically Black College or University (HBCU) and later-life cognition, which included demographics, birth cohort, and early-life schooling, socioeconomic status (SES), health, and area-level exposures. To ensure that exposure groups had similar probabilities of attending an HBCU, the primary analysis was conditioned on those who attended high school in a state with an HBCU.

© 2026 Thomas MD et al. *JAMA Network Open*.

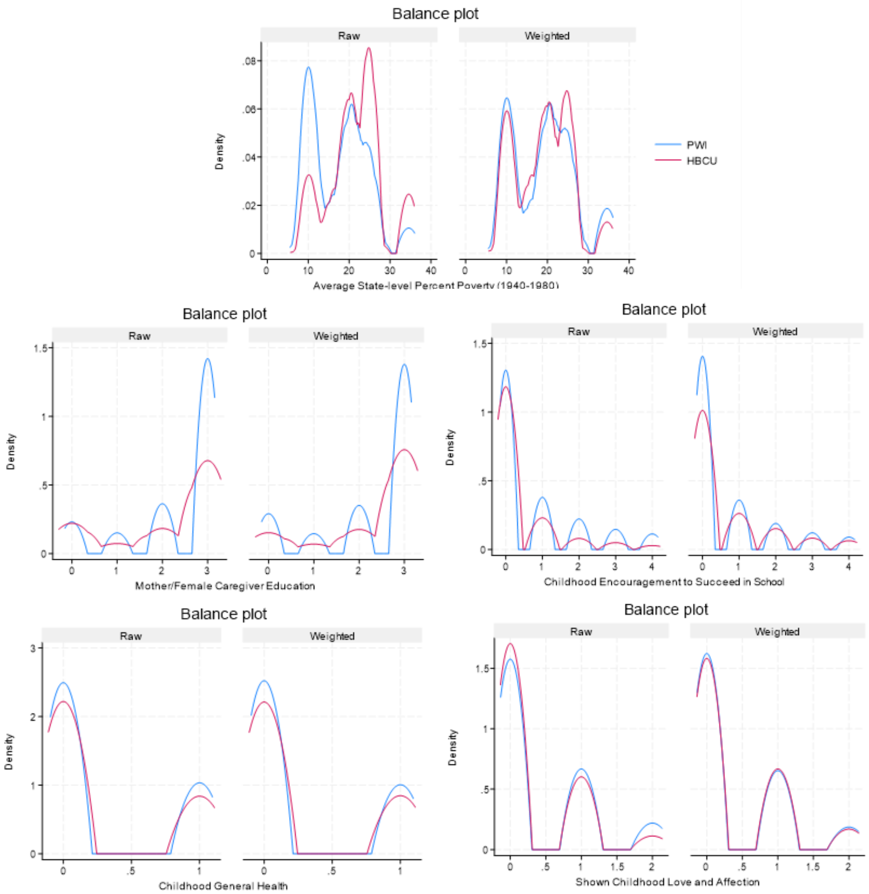

eFigure 2a. The distribution of selected covariates between treatment groups in the reweighted population. The x-axis represents the categorial or numeric covariate values. The y-axis represents kernel density estimates. Each plot shows the raw distribution on the left and the weighted distribution on the right. PWI attendees are shown in blue. HBCU are shown in red.

	Memory				Language			
	Raw		Weighted		Raw		Weighted	
Total Observations	2133		2133		2154		2154	
Treated Observations	657		1057		663		1067	
Control Observations	1476		1075		1491		1088	
	Standardized Difference		Variance Ratio		Standardized Difference		Variance Ratio	
	Raw	*Weighted*	*Raw*	*Weighted*	*Raw*	*Weighted*	*Raw*	*Weighted*
**Assessment Age**	0.140	0.001	0.939	0.890	0.130	0.002	0.960	0.950
**Gender**	-0.030	-0.028	1.024	1.021	-0.028	-0.030	1.022	1.022
**College Age**								
'ost-*Brown*/Pre-CRA (1955 - 1964)	0.014	-0.001	1.003	1.000	0.027	-0.002	1.005	1.000
Post-CRA (> 1964)	-0.128	-0.003	0.871	0.997	-0.130	0.000	0.868	1.000
**Community size at birth**	0.085	0.006	0.965	0.997	0.081	0.006	0.967	0.998
**Mother's/Caregiver's education**								
Post HS Vocational/Professional	-0.023	-0.028	0.923	0.903	-0.026	-0.030	0.915	0.898
HS/GED	-0.032	-0.027	0.943	0.950	-0.025	-0.027	0.956	0.951
<HS/None/Unknown	-0.140	0.039	1.074	0.977	-0.143	0.040	1.075	0.976
**Childhood encouragement to succeed in school**								
Most of the time	-0.078	0.002	0.866	1.003	-0.078	0.002	0.866	1.004
Some of the time	-0.193	0.031	0.532	1.093	-0.193	0.032	0.529	1.094
Little of time	-0.168	-0.017	0.486	0.937	-0.166	-0.016	0.486	0.939
None of the time	-0.185	-0.002	0.363	0.993	-0.186	-0.004	0.359	0.981
**Childhood general health**	-0.040	-0.022	0.964	0.979	-0.041	-0.019	0.962	0.982
**Shown childhood love and affection**								
Some/Most the time	-0.055	0.024	0.943	1.024	-0.050	0.024	0.947	1.025
Little/None/Unknown time	-0.161	-0.020	0.564	0.935	-0.169	-0.022	0.549	0.930
**% Poverty**	0.565	0.012	0.915	0.834	0.558	0.013	0.908	0.825

Abbreviations: CRA = Civil Rights Act, HS = high school, GED = general education diploma

eFigure 2b. The estimates for covariate balance by treatment group after estimation of the inverse probability-weighted estimator. The table on the left was generated using memory as the outcome. The table on the right was generated using language as the outcome.

eFigure 2. Postestimation covariate balance results prior to multiple imputation.

© 2026 Thomas MD et al. *JAMA Network Open*.

eTable 1. Associations between covariates within each childhood domain

Associations by Covariate Domain	chi-square value	p-value
**Socioeconomic Status**		
male and female caregivers' education	1100.000	<0.001
female caregivers' education and house type	37.554	<0.001
female caregivers' education and home ownership	238.645	<0.001
female caregivers' education and land ownership	95.349	<0.001
male caregivers' education and house type	31.001	<0.001
male caregivers' education and home ownership	155.545	<0.001
male caregivers' education and land ownership	40.963	<0.001
**Academics**		
homework help and encouragement to succeed	1900.000	<0.001
**Health**		
general health and birth weight	31.969	<0.001
general health and days of hunger	100.547	<0.001
birth weight and days of hunger	0.447	0.978
**Social Support**		
parent in jail and death of a parent	13.627	<0.001
parent in jail and witnessed domestic violence	349.202	<0.001
shown love/affection and witnessed domestic violence	247.689	<0.001
shown love/affection and death of parent	25.407	<0.001
shown love/affection and parent in jail	61.771	<0.001
**Area-level (%)**	**Pearson correlation value**	**p-value**
Black population - Hispanic population	-0.387	<0.10
Black population - foreign born	-0.470	<0.10
Black population - below federal poverty	0.701	<0.10
Hispanic population - foreign born	0.591	<0.10
Hispanic population - below federal poverty	-0.319	<0.10
below federal poverty - foreign born	-0.675	<0.10

© 2026 Thomas MD et al. *JAMA Network Open*.

eTable 2. Bivariable associations of covariates with memory, language, and HBCU status

Variable	Memory-Covariate Association		Language-Covariate Association		HBCU-Covariate Association	
	F † or T statistic	p-value	F † or T statistic	p-value	chi-square value	p-value
**Socioeconomic Status**						
dad/male caregiver education †	24.210	<0.001	18.890	<0.001	31.502	<0.001
mom/female caregiver education †	19.320	<0.001	15.130	<0.001	82.379	<0.001
type of housing †	2.610	0.740	0.010	0.985	39.624	<0.001
land ownership	0.859	0.390	-0.827	0.409	25.760	<0.001
home ownership †	13.320	<0.001	9.560	0.001	64.760	<0.001
**Academics**						
homework help †	8.260	0.000	1.990	0.137	56.732	<0.001
encouragement to succeed †	4.960	0.001	2.710	0.137	118.640	<0.001
**Health**						
general health †	33.510	< 0.001	0.480	0.487	0.619	0.432
birth weight †	3.160	0.042	2.140	0.118	0.596	0.742
days of hunger †	8.420	0.000	0.260	0.773	20.708	< 0.001
**Social Support**						
shown love and affection †	2.370	0.005	0.290	0.750	23.728	< 0.001
parental death	3.961	< 0.001	3.011	0.003	4.219	0.040
witnessed domestic violence	-1.318	0.188	-1.779	0.076	28.077	< 0.001
**State-level**	**Pearson Correlation**	**p-value**	**Pearson Correlation**	**p-value**	**T-statistic**	**p-value**
Black population	-0.009	> 0.10	-0.065	< 0.10	-16.219	< 0.001
Hispanic population	0.004	> 0.10	0.007	> 0.10	10.091	< 0.001
foreign born	0.062	< 0.10	0.058	< 0.10	16.626	< 0.001
below federal poverty	-0.063	< 0.10	0.093	< 0.10	-17.059	< 0.001

Abbreviations: HBCU = historically Black College or University

© 2026 Thomas MD et al. *JAMA Network Open*.

eTable 3. State or region with a historically Black college or university (HBCU)

Alabama
Arkansas
Delaware
District of Columbia
Florida
Georgia
Kentucky
Louisiana
Maryland
Mississippi
Missouri
North Carolina
Ohio
Oklahoma
Pennsylvania
South Carolina
Tennessee
Texas
U.S. Virgin Islands
Virginia
West Virginia

Source: https://nces.ed.gov/programs/digest/d23/tables/dt23_313.10.asp

© 2026 Thomas MD et al. *JAMA Network Open*.

eTable 4 (Illustrated in Figures 3 & 4): Primary estimates of differences between HBCU versus PWI attendees on cognition at mean age 62 among Black adults in REGARDS, and estimates stratified by college-aged exposure to legal racial segregation (Pre-Brown) and Civil Rights Act (CRA)

Outcome	N	ATE	95% CI	p-value
**MEMORY**				
**Primary Analysis**	**1952**	**0.13**	**(0.05, 0.21)**	**0.001**
**Stratified by College-aged Cohort**				
Pre-*Brown* (< 1955)	513	0.12	(-0.05, 0.28)	0.164
Post-*Brown*/Pre-CRA (1955 - 1964)	884	0.10	(0.02, 0.17)	0.010
Post-CRA (> 1964)	555	0.13	(-0.05, 0.31)	0.162
**LANGUAGE**				
**Primary Analysis**	**1970**	**0.19**	**(0.08, 0.29)**	**< 0.001**
**Stratified by College-aged Cohort**				
Pre-*Brown* (< 1955)	516	0.20	(-0.01, 0.41)	0.065
Post-*Brown*/Pre-CRA (1955 - 1964)	894	0.20	(0.07, 0.33)	0.003
Post-CRA (> 1964)	560	0.14	(0.03, 0.26)	0.017
**GLOBAL COGNITION**				
**Primary Analysis**	**530**	**0.22**	**(0.09, 0.34)**	**0.001**
**Stratified by College-aged Cohort**				
Pre-*Brown* (< 1955)	103	0.23	(0.00, 0.58)	0.050
Post-*Brown*/Pre-CRA (1955 - 1964)	221	0.12	(-0.02, 0.26)	0.083
Post-CRA (> 1964)	206	0.33	(0.16, 0.51)	< 0.001

Abbreviations: ATE = average treatment effect using inverse of probability of treatment weighting, CI = confidence interval, CRA = Civil Rights Act
Notes: Cognitive measures are z-score transformed (i.e., standardized). Linear regression models estimate the average treatment effect of HBCU attendance as differences in standard deviation using inverse probability of treatment weighting. Primary analysis models are adjusted for college-age birth cohort, age at assessment, gender, community size at birth, mom's/caregiver's education, and childhood encouragement to succeed in school, general health, love and affection shown, and state percent in federal poverty. Effect modification models are adjusted for the same covariates except within strata of birth cohort.

© 2026 Thomas MD et al. *JAMA Network Open*.

eTable 5 (Sensitivity Results). Estimates of the association between HBCU versus PWI Attendance on Cognition among Black Adults at Mean Age 62 in REGARDS

MODEL	N	ATE	95% CI	p-value
**MEMORY**				
**IPTW**				
**HBCU states (Primary Analysis)**	**1952**	**0.13**	**(0.05, 0.21)**	**0.001**
All states	2712	0.12	(0.05, 0.19)	0.001
HBCU states, add state fixed effects	1952	0.13	(0.06, 0.21)	0.001
HBCU states, adjust for % Black pop	1690	0.10	(0.02, 0.18)	0.019
HBCU states, adjust for % Black pop and % poverty	1690	0.11	(0.03, 0.19)	0.006
HBCU states, not adjusted for state-level variables	1952	0.13	(0.04, 0.21)	0.004
HBCU states, outliers removed	1952	0.13	(0.05, 0.21)	0.001
**Linear Regression**				
HBCU states	1952	0.15	(0.07, 0.23)	0.001
All States	2712	0.13	(0.06, 0.20)	0.000
HBCU states, add state fixed effects	1952	0.14	(0.06, 0.23)	0.003
**LANGUAGE**				
**IPTW**				
**HBCU states (Primary Analysis)**	**1970**	**0.19**	**(0.08, 0.29)**	**< 0.001**
All states	2738	0.16	(0.07, 0.25)	< 0.001
HBCU states, add state fixed effects	1970	0.20	(0.09, 0.30)	< 0.001
HBCU states, adjust for % Black pop	1705	0.18	(0.07, 0.28)	0.001
HBCU states, adjust for % Black pop and % poverty	1705	0.19	(0.08, 0.30)	0.001
HBCU states, not adjusted for state-level variables	1970	0.17	(0.07, 0.27)	0.001
HBCU states, outliers removed	1947	0.17	(0.09, 0.25)	< 0.001
**Linear Regression**				
HBCU states	1970	0.21	(0.08, 0.34)	0.003
All States	2738	0.18	(0.09, 0.28)	0.001
HBCU states, add state fixed effects	1970	0.22	(0.09, 0.35)	0.003

GLOBAL COGNITION

© 2026 Thomas MD et al. *JAMA Network Open*.

**IPTW**				
**HBCU states (Primary Analysis)**	**530**	**0.22**	**(0.09, 0.34)**	**< 0.001**
All states	720	0.18	(0.06, 0.29)	0.002
HBCU states, add state fixed effects	530	0.24	(0.11, 0.36)	< 0.001
HBCU states, adjust for % Black pop	462	0.18	(0.06, 0.29)	0.002
HBCU states, adjust for % Black pop and % poverty	462	0.19	(0.07, 0.30)	0.001
HBCU states, not adjusted for state-level variables	530	0.21	(0.09, 0.34)	0.001
HBCU states, outliers removed	525	0.21	(0.10, 0.32)	< 0.001
**Linear Regression**				
HBCU states	530	0.21	(0.03, 0.38)	0.024
All States	720	0.17	(0.03, 0.30)	0.020
HBCU states, add state fixed effects	530	0.21	(0.04, 0.39)	0.022

Abbreviations: HBCU = historically Black college or university, PWI = predominantly White institution, CI = confidence interval, ATE = average treatment effect using inverse of probability of treatment weighting, IPTW = inverse probability of treatment weighting, Pop = population

Notes: Notes: Cognitive measures are z-score transformed (i.e., standardized). Linear regression models estimate the average treatment effect of HBCU attendance as differences in standard deviation using inverse probability of treatment weighting. Models are adjusted for college-age birth cohort, age at assessment, gender, community size at birth, mom's/caregiver's education, and childhood encouragement to succeed in school, general health, love and affection shown, and state percent in federal poverty (except the average treatment effect model adjusting or percent Black population does not adjust for poverty).

© 2026 Thomas MD et al. *JAMA Network Open*.
