# Supplementary material for: Historically Black College or University Attendance and Cognition in US Black Adults
Source: JAMA Netw Open. 2026 Feb 11;9(2):e2558329. doi: 10.1001/jamanetworkopen.2025.58329 (PMC12895292; doi:10.1001/jamanetworkopen.2025.58329)
Supplement: Supplement 2. — Data Sharing Statement [file jamanetwopen-e2558329-s002.pdf]

Data Sharing Statement

Thomas. Historically Black College or University Attendance and Cognition in US Black Adults. *JAMA Netw Open*. Published February 11, 2026. doi:10.1001/jamanetworkopen.2025.58329

Data
Data available: No

Additional Information
Explanation for why data not available: We used restricted use data and do not have permission to share. However, a data dictionary is available online. We can make our derived data dictionary available as well as our programming code.
